# Atopic sensitization to common allergens without symptoms or signs of airway disorders does not increase exhaled nitric oxide

**DOI:** 10.1111/j.1752-699X.2007.00045.x

**Published:** 2008-07

**Authors:** Annamari Rouhos, Annette Kainu, Jouko Karjalainen, Ari Lindqvist, Päivi Piirilä, Seppo Sarna, Tari Haahtela, Anssi R A Sovijärvi

**Affiliations:** 1 Division of Respiratory Diseases, Department of Medicine, Helsinki University Central HospitalHelsinki, Finland; 2 Research Unit for Pulmonary Diseases, Clinical Research Institute Ltd, Helsinki University Central HospitalHelsinki, Finland; 3 Division of Allergology, Department of Medicine, Helsinki University Central HospitalHelsinki, Finland; 4 Division of Clinical Physiology and Nuclear Medicine, Laboratory Department, Helsinki University Central HospitalHelsinki, Finland; 5 Institute of Military MedicineHelsinki, Finland; 6 Department of Public Health, Helsinki UniversityHelsinki, Finland

**Keywords:** airway inflammation, atopy, exhaled nitric oxide, healthy adults, skin prick tests

## Abstract

**Background:**

Elevated fractional exhaled nitric oxide (FENO) associates positively with symptomatic atopy among asthmatics and in the general population. It is, however, unclear whether sensitization to common allergens *per se*– as verified with positive skin prick tests – affects FENO in healthy individuals.

**Objective:**

The aim of this study was to examine the association between FENO and sensitization to common allergens in healthy nonsmoking adults with no signs or symptoms of airway disorders.

**Methods:**

FENO measurements (flow rate: 50 mL/s), skin prick tests to common inhalant allergens, structured interviews, spirometry, bronchodilatation tests and bronchial histamine challenges were performed on a randomly selected population of 248 subjects. Seventy-three of them (29%) were nonsmoking asymptomatic adults with no history of asthma, persistent or recurrent upper or lower airway symptoms and no signs of airway disorders in the tests listed above.

**Results:**

FENO concentrations were similar in skin prick test positive (*n* = 32) and negative (*n* = 41) healthy subjects, with median values of 13.2 and 15.5 ppb, respectively (*P* = 0.304). No correlation appeared between FENO and the number of positive reactions (*r* = −0.138; *P* = 0.244), or the total sum of wheal diameters (*r* = −0.135; *P* = 0.254). The nonparametric one-tailed 95% upper limits of FENO among skin prick positive and negative healthy nonsmoking subjects were 29 and 31 ppb, respectively.

**Conclusions:**

Atopic constitution defined as positive skin prick test results does not increase FENO in healthy nonsmoking adults with no signs or symptoms of airway disorders. This suggests that same reference ranges for FENO can be applied to both skin prick test positive and negative subjects.

Please cite this paper as: Rouhos A, Kainu A, Karjalainen J, Lindqvist A, Piirilä P, Sarna S, Haahtela T and Sovijärvi ARA. Atopic sensitization to common allergens without symptoms or signs of airway disorders does not increase exhaled nitric oxide. *The Clinical Respiratory Journal* 2008; 2: 141–148.

## Introduction

Fractional exhaled nitric oxide (FENO) is a useful, noninvasive marker for assessment of eosinophilic airway inflammation (1). It is elevated in patients with asthma-like symptoms (2) and in asthma (2, 3), especially in atopic asthma (4). FENO rapidly decreases with anti-inflammatory therapy (1), and increases again with deterioration of asthma control (1). FENO measurement may serve as a useful tool in the diagnosis of asthma and in the early detection of eosinophilic airway inflammation (1, 5), as well as in guiding anti-inflammatory treatment of asthma (6).

Many studies have addressed the effect of clinical atopic conditions on FENO. While FENO levels have been higher in atopic than in nonatopic asthma, it is unclear whether healthy atopic subjects without symptoms or signs of airway disorders also have higher FENO levels than nonatopic subjects (7–13). Studies based on general population samples have generally found FENO to be higher in atopic than in nonatopic subjects (8, 11, 13). Variable results may be explained by differences in study populations, definition of atopy and in the expiratory flow rates used in FENO measurements. Two recent landmark studies (12, 13) define reference ranges of FENO for adults, but leave open the question of whether reference populations could include healthy subjects regardless of their atopic constitution.

The aim of the present study was to examine the association of FENO and skin prick test results in healthy nonsmoking adults with no symptoms or signs of airway disorders assessed by structured interviews, spirometry and bronchial challenge tests.

## Materials and methods

### Study population and design

The present study population originated from the FinEsS study, which is a large epidemiological investigation on obstructive lung diseases, respiratory symptoms and type I allergies carried out in Finland (Fin), Estonia (Es) and Sweden (S). As part of FinEsS, a postal questionnaire was sent in 1996 to 8000 20- to 69-year-old inhabitants of Helsinki. The study sample was obtained from the Population Register Center, randomized by 10-year age cohorts and by gender. The results from that study have been reported previously (14). Of the 6062 postal survey responders, 1200 subjects were later randomly selected and invited for clinical studies (spirometry, bronchodilatation tests, skin prick tests and structured interviews). Half (600) of them were at the same time further randomized to FENO measurements and histamine challenges. Of the 600 invited, 310 (51.7%) participated in the study.

The 310 subjects, aged 26–65 years, underwent on the first study day a structured interview, flow-volume spirometry and a bronchodilatation test as explained later. Skin prick tests were performed on the same day on those younger than 61 years of age. FENO measurements and histamine challenges, in that order, were performed on the second study day, which was 1–14 days after the first session. The measurements were carried out throughout the period from May 2001 to March 2003.

**Figure 1:**
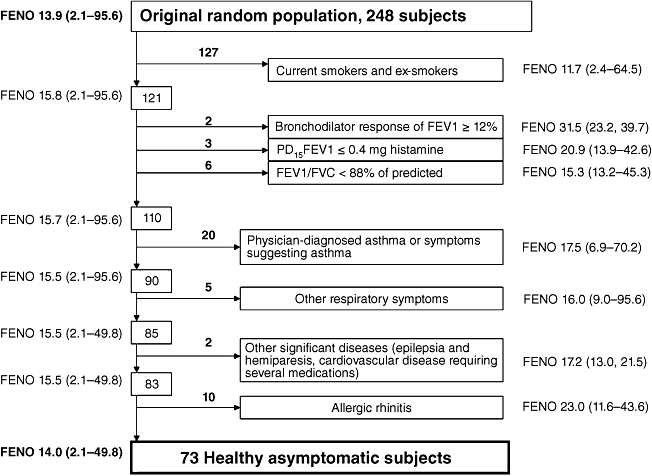
Exclusion process to select healthy asymptomatic nonsmoking subjects, with fractional exhaled nitric oxide (FENO) median and range shown for each subgroup.

A technically acceptable FENO measurement was obtained from 295 subjects, with skin prick test data available from 248 of them (84%). From this population, subjects were included in the present comparative analysis, provided they had: (i) no significant smoking history (nonsmokers or ex-smokers with a history of <5 pack-years and smoking cessation >5 years previously); (ii) no signs of airway obstruction [forced expiratory volume in 1 s (FEV1)/forced vital capacity (FVC) ≥ 88% of predicted (15), and bronchodilator response < 12% increase in FEV1]; (iii) no significant bronchial hyperresponsiveness (PD_15_FEV1 > 0.4 mg histamine (16)); (iv) no previously diagnosed asthma, chronic bronchitis or chronic obstructive pulmonary disease, chronic or recurrent symptoms from the upper or lower respiratory tracts (cough, sputum production, wheezing, shortness of breath, nasal symptoms related to specific allergens); or (v) no symptoms of cardiovascular, gastrointestinal or neurological diseases. Obesity as such was not regarded as an exclusion criterion. Furthermore, the subjects had to be free from acute respiratory infections for the 3 weeks prior to the study. After that exclusion process, the remaining 73, out of the 248 subjects, formed the study population of healthy asymptomatic nonsmoking adults (Fig. 1). The characteristics of the original random population of 248 subjects and of the further analyzed 73 subjects are given in Table 1. No differences appeared between skin prick test positive and negative healthy subjects in relation to anthropometric or spirometric variables.

**Table 1:** Characteristics of the studied populations

		All subjects (*n* = 248)	Healthy subjects (*n* = 73)	Skin prick test positive (*n* = 32)	Skin prick test negative (*n* = 41)
Gender	Male/Female	106/142	27/46	11/21	16/25
Atopy	Atopics/Nonatopics	116/132	32/41	32/0	0/41
Smoking history	Non-/Ex-/Current smokers	121/38/89	73/0/0	32/0/0	41/0/0
Age (years)	Mean (range)	45 (26–61)	44 (27–61)	43 (27–61)	45 (28–61)
Height (cm)	Mean (range)	170.5 (146–198)	170.8 (155–189)	170.3 (156–189)	171.3 (155–188)
Weight (kg)	Mean (range)	74.5 (45–139)	71.8 (45–111)	70.7 (45–96)	72.6 (56–111)
Body mass index	Mean (range)	25.5 (17.1–53.3)	24.5 (18.5–38.9)	24.3 (18.5–34.0)	24.7 (19.3–38.9)
FVC (L)	Mean (range)	4.4 (2.2–7.5)	4.4 (2.7–6.3)	4.4 (2.7–6.3)	4.4 (3.0–6.2)
FVC, % of predicted*	Mean (range)	98.1 (67–145)	99.4 (72–145)	99.4 (72–125)	99.3 (76–145)
FEV1 (L)	Mean (range)	3.4 (1.0–5.6)	3.5 (2.4–5.4)	3.5 (2.4–5.0)	3.5 (2.4–5.4)
FEV1, % of predicted*	Mean (range)	93.0 (41–129)	97.3 (72–129)	96.9 (75–127)	97.6 (72–129)
FEV1/FVC, % of predicted*	Mean (range)	94.9 (55–115)	98.2 (88–115)	97.7 (88–109)	98.5 (90–115)

* Finnish reference values (15).

FVC, forced vital capacity; FEV1, forced expiratory volume in 1 s.

### FENO measurement

FENO was measured with a chemiluminescence analyzer (Sievers 270B; Boulder, CO, USA). Two-point calibration of the analyzer was performed daily before FENO measurements were taken. The expiratory airflow and exhaled volume were measured with a pneumotachograph (Baby Pneumotachograph, Erich Jaeger GmbH, Wurzburg, Germany) simultaneously with FENO in real time. The exhalation procedure fulfilled the criteria defined in the European Respiratory Society/American Thoracic Society (ATS) guidelines on exhaled FENO measurements (17). Before the measurement, subjects rinsed their mouths with sodium bicarbonate solution (Hartwall Novelle®; Oy Hartwall AB, Helsinki, Finland) to eliminate any nitric oxide eventually produced in the mouth. After inhalation of NO-free gas (100% oxygen), subjects exhaled from total lung capacity with a flow rate of 50 mL/s against a flow resistor (Hans Rudolph, Shawnee, KS, USA; model #7100R, 200 cm H_2_O/L/s, flow range: 0–0.1 L/s) to close the soft palate, thus avoiding any nasal NO contamination. No nose clips were used. The subjects maintained the required flow rate with the aid of a visual feedback from the computer screen. The mean flow rate for acceptable measurement was between 0.045 and 0.055 L/s, and the duration of exhalation was at least 10 s. The mean value taken from a 3-s period from the end-exhaled NO plateau was recorded for analysis. At least three successive FENO measurements were performed, and their mean value was recorded for analysis. The acceptable coefficient of variation of the successive FENO determinations was <0.15 (mean 0.05).

### Skin prick testing

The skin prick tests were performed with 15 common aeroallergens (cat, dog, cow, horse, birch, timothy, mugwort, the house dust mites *Dermatophagoides pteronyssinus* and *Dermatophagoides farinae*, the storage mites *Acarus siro* and *Lepidoglyphus destructor*, the outdoor molds *Cladosporium* and *Alternaria*, cockroach and latex) with histamine dihydrochloride (10 mg/mL) as the positive, and the solvent (glycerol) as the negative control. All extracts were provided by ALK, Hørsholm, Denmark, except latex, which was provided by Alyostal ST-IR, Paris, France. The tests were carried out by three experienced study nurses on the volar side of the forearm. The reactions were inspected after 15 min, and the wheal size was measured in millimeters in two perpendicular directions including the longest diameter with their mean recorded as the response. A response of ≥3 mm in the presence of expected results to the control solutions was regarded as positive (18). Subjects with at least one positive reaction were regarded as skin prick test positive. Because of decreasing reactivity of the skin with increasing age, subjects over 60 years of age did not undergo skin prick testing (19).

### Spirometry

The FVC and FEV1 were measured according to ATS recommendations (20), using a flow-volume spirometer (SensorMedix Vmax 20C; Yorba Linda, CA, USA). For bronchodilatation tests, 0.4 mg of inhaled salbutamol aerosol (Ventoline®; GlaxoSmithKline, Brentford, UK) was administered via a spacer (Volumatic®; GlaxoSmithKline). FVC and FEV1 measurements were repeated 15 min after the administration of salbutamol. The results are expressed as absolute values and as a percentage of the predicted value with Finnish reference values used (15).

### Histamine challenge

The histamine challenge for assessment of bronchial responsiveness was performed by a dosimetric method with controlled tidal breathing (16) after FENO measurement. A provocative dose of histamine inducing a 15% decrease of FEV1 (PD_15_FEV1) was calculated from the logarithmically transformed histamine doses by use of linear interpolation.

### Structured interview

The structured interview was conducted by one of the five physicians involved in this part of the study, using a questionnaire developed for the FinEsS studies. The origins of the questionnaire have been reported previously (21). The questionnaire included detailed questions on recent or past respiratory symptoms, diagnosed asthma or other chronic respiratory diseases, history of cardiovascular diseases or other major diseases, medication used and smoking history.

### Statistical analysis

Statistical analyses were performed using SPSS version 11.0 for Windows (SPSS, Chicago, IL, USA). As FENO was not normally distributed, the results are expressed as median values and 25%–75% quartile ranges. Comparisons of median FENO values between the groups were analyzed with the Mann–Whitney *U*-test. Comparisons of anthropometric and spirometric variables between atopic and nonatopic healthy subjects were performed using an independent sample *t*-test. Mutual correlations between FENO and the number of positive skin prick tests, between FENO and the total sum of wheal diameters as well as between FENO and age, weight and height were analyzed with Spearman's rank correlation coefficients. All tests were two-sided, and a *P* value of <0.05 was considered significant. As FENO was not normally distributed and normal distribution was not achieved by log transformation, a nonparametric ranking system was used for assessing the one-tailed 95% upper limit in the groups.

## Results

Results from structured interviews, FENO measurements, skin prick tests, spirometry and bronchodilatation tests were available from all 73 subjects fulfilling the inclusion criteria of healthy asymptomatic nonsmokers, as well as from the 175 excluded subjects. Histamine challenge results were available from 71 included subjects (two subjects refused) and from 168 excluded subjects. Three subjects had mild restrictive ventilation impairment according to FVC and FEV1 values (scoliosis, obesity and individual body structure [body mass index (BMI) 18]. The impact of the exclusion process on median FENO values and FENO ranges is shown in Fig. 1.

**Figure 2:**
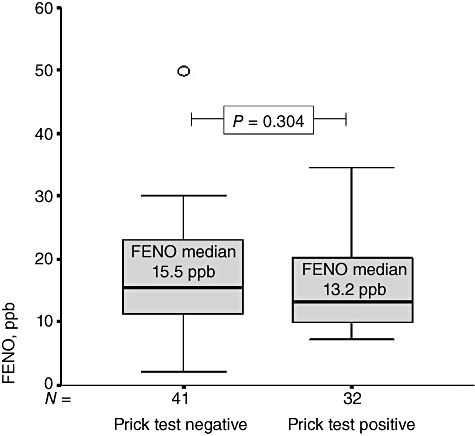
Fractional exhaled nitric oxide (FENO) in healthy asymptomatic skin prick positive and negative subjects. Data are expressed as medians with interquartile range (box) and range (whiskers) excluding an outlier (circle).

**Figure 3:**
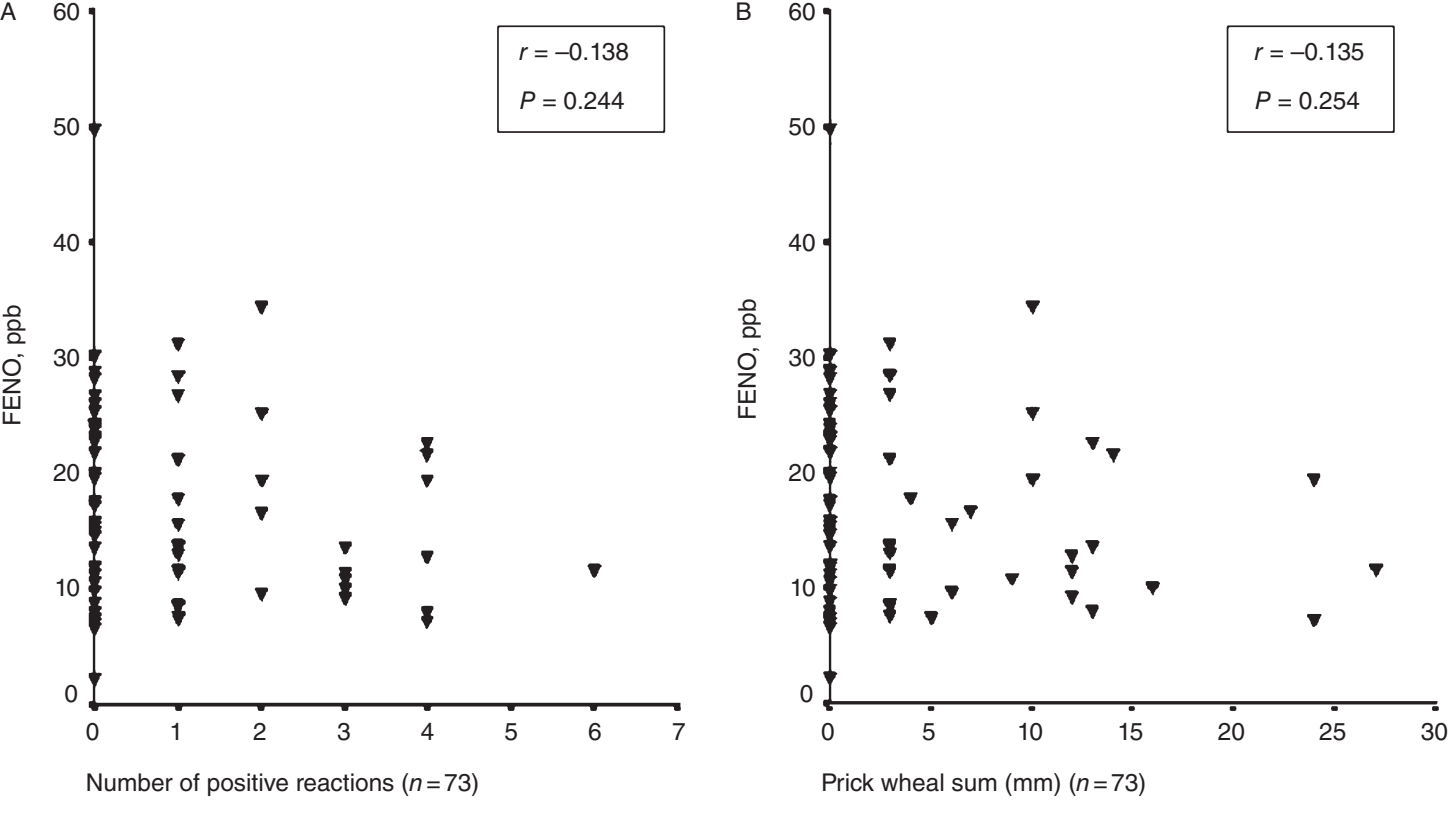
Correlation between fractional exhaled nitric oxide (FENO) and the number of positive skin prick tests (A) and between FENO and the total prick wheal sum (calculated by adding the diameters of each positive reaction) (B) in healthy nonsmoking adults with no signs or symptoms of airway disease (*n* = 73).

Median FENO in the 73 healthy asymptomatic nonsmoking subjects was 15.0 ppb. Median FENO was similar in skin prick positive (*n* = 32) and skin prick negative (*n* = 41) subjects, 13.2 and 15.5 ppb,respectively (Fig. 2). No significant correlation appeared between FENO and the degree of atopy defined as the number of positive reactions or as the total sum of wheal diameters (calculated by adding the diameters of each positive reaction (≥3 mm) (Fig. 3). In contrast, among those subjects (*n* = 175) who were excluded because of history, symptoms or signs of respiratory disease or smoking, a significant correlation was observed between FENO and the number of positive reactions (*r* = 0.160; *P* = 0.034) or the total sum of wheal diameters (*r* = 0.178; *P* = 0.019), as well as in the subpopulation of nonsmoking excluded subjects (*n* = 48) (*r* = 0.345; *P* = 0.016) and (*r* = 0.343; *P* = 0.017), respectively. Mean wheal size and the total sum of wheal diameters were significantly higher in excluded subjects compared with healthy subjects (4.9 and 3.8 mm, respectively (*P* = 0.005), and 14.1 and 8.8, respectively (*P* = 0.047). The number of positive reactions was similar in these groups (2.2 and 2.8, respectively; *P* = 0.25). Of the healthy skin prick positive subjects, 44% were sensitized to only perennial allergens, 31% to only seasonal allergens and 25% to both perennial and seasonal allergens, whereas among excluded skin prick positive subjects, the percentages were 18%, 39% and 43%, respectively.

No significant correlation appeared between FENO and age (*r* = 0.025; *P* = 0.833), FENO and weight (*r* = 0.222; *P* = 0.059) or FENO and BMI (*r* = 0.152; *P* = 0.198), but a correlation did appear between FENO and height (*r* = 0.267; *P* = 0.022). The one-tailed nonparametric 95% upper limit for FENO in the whole group of healthy asymptomatic nonsmoking subjects was 30 ppb, among the skin prick positive healthy subjects it was 31 ppb and among skin prick test negative healthy subjects it was 29 ppb.

## Discussion

We observed similar levels of FENO in skin prick positive and negative healthy asymptomatic nonsmoking adults with no signs of airway disorders, indicating that atopic sensitization *per se* does not elevate FENO. Horváth and Barnes found higher levels of FENO in atopic than in nonatopic healthy asymptomatic subjects (22). Their study population, however, included only 15 atopic subjects, most of whom had mild to moderate bronchial hyperresponsiveness on methacholine challenge. Also, Franklin *et al.*(8) found higher FENO levels in atopic than in nonatopic subjects in a heterogeneous study population of 115 subjects. Even though all subjects were asymptomatic at the time of the study, 26% had physician-diagnosed asthma, 17% reported wheezing during the previous year and 17% had increased bronchial responsiveness. Olin *et al.*(7) found similar levels of FENO in 137 skin prick positive and 33 skin prick negative healthy adults. Their study population was not a random sample of healthy adults because all subjects had occupational low-level irritant exposure as bleachery workers.

More recently, Olin *et al.*(11) found atopy being a significant predictor of FENO in the large general population even after exclusion of subjects with asthma or current asthma symptoms during the previous month. However, subjects with earlier history of chronic or recurrent respiratory symptoms were not excluded from the analyses, which may affect the results. In their study, atopy was assessed by measuring total serum IgE, which has significant variation by gender, age and smoking, as well as considerable overlapping of normal and atopic ranges (23).

We found no significant correlation between FENO and the degree of atopy defined either as the number of positive reactions or as the total sum of wheal diameters among healthy nonsmoking subjects. This is not necessarily controversial with the studies by Ho *et al.*(24), or by Franklin *et al.*(8), where significant correlation appeared between FENO and the number of positive skin prick tests, because both studies included subjects with physician-diagnosed asthma or even recent respiratory symptoms suggesting asthma. In the present study, a positive correlation between FENO and the degree of atopy did appear among the excluded subjects, i.e. subjects with a history or with symptoms or signs of airway disorder. Excluded subjects were sensitized to equal numbers of allergens as the healthy subjects, but their sensitization was more intensive, and the type of sensitization differed from that in healthy subjects.

Patients with asthma-like symptoms may have increased FENO (2), and subclinical inflammation can be present in patients with asthma in remission, resulting in elevated FENO (25). Although subjects with positive skin prick tests are about three times more likely to develop asthma (26), atopic constitution defined as positive reactions in skin prick tests or as elevated allergen-specific IgE levels in serum, does not imply presence of a disease or active inflammation. Up to 39% of skin prick positive subjects from a general adolescent population have been reported to be asymptomatic (27).

Prieto *et al.* found increasing FENO levels when patients with allergic rhinitis were studied during the pollen season (28), i.e. the mucosal inflammation was activated. In our study, FENO was measured throughout the year, and no significant difference occurred between FENO levels measured during or out of the pollen season. However, the only eight FENO measurements were performed on atopic subjects during the pollen season compared with 24 measurements performed out of the pollen season, thus not allowing conclusive comparison.

Our study population of nonsmoking healthy asymptomatic adults selected from a random population is most relevant for assessment of influence of atopic constitution on FENO. Possible airway pathology even in asymptomatic subjects was taken into account by excluding subjects with abnormal spirometry or bronchial challenge. Atopy was assessed objectively according to published guidelines (18), based on skin prick tests for a large number of common aeroallergens. To our knowledge, this is the largest study of such a random healthy population comparing FENO between skin prick positive and negative subjects. The 27- to 61-year age group represents well the target adult population for FENO measurements in a clinical setting. Because smoking is known to reduce the level of FENO, current smokers were not included in the study. Even though the effect of smoking wanes within weeks after smoking cessation (29), smoking can cause more chronic detrimental effects on the airway mucosa, which might influence FENO production. Thus, in addition to nonsmokers, we included only ex-smokers with very short smoking histories and several years since smoking cessation.

Reference values based on gender and atopic status were suggested by the recent study by Travers *et al.*(13). Their study excluded subjects with respiratory symptoms within 12 months or physician-diagnosed asthma and symptoms or inhaler use within 12 months, as well as those with reversible airway obstruction in lung function. These criteria, however, do not exclude subjects with mild intermittent asthma, who often may go undiagnosed and are reported to have elevated FENO as a sign of ongoing eosinophilic inflammation in the airways (30). The definition of atopy was based on skin prick tests performed with nine common allergens, but the procedure, the criteria for positive result and the solutions used, were not described (13). Previously, large differences in the prevalence of positive reactions have been observed with different commercial preparations of the same allergen (31). A very recent study by Olin *et al.*(12) suggested reference values of FENO for adults to be adjusted according to height and age, whereas atopic status, defined as increased levels of serum IgE, was found to be of minor importance, thus suggesting same upper normal limits for both atopic and nonatopic healthy nonsmoking subjects. Our study found a significant correlation between FENO and height, but not with FENO and age. The upper 95% limit of FENO, 29–31 ppb, reported in the present study is in line with that of 24–53 ppb suggested in the study by Olin *et al.*(12) as well as with that of 33 ppb suggested by Taylor *et al.* based on earlier data (1).

Our findings indicate that in healthy asymptomatic nonsmoking adults, atopic constitution (positive skin prick test results) *per se* does not influence FENO. We conclude further that elevated FENO is not an indicator of atopic constitution, but indicates NO-producing inflammation in the airways. These findings suggest that same reference range can be applied to both skin prick positive and negative subjects.
